# Production of erythriod cells from human embryonic stem cells by fetal liver cell extract treatment

**DOI:** 10.1186/1471-213X-10-85

**Published:** 2010-08-10

**Authors:** Yu-xiao Liu, Wen Yue, Lei Ji, Xue Nan, Xue-tao Pei

**Affiliations:** 1 Stem Cell and Regenerative Medicine Lab, Beijing Institution of Transfusion Medicine, Beijing 100850, China; 2 Department of Neurosurgery, the First Affiliated Hospital of PLA General Hospital, Beijing 100037, China

## Abstract

**Background:**

We recently developed a new method to induce human stem cells (hESCs) differentiation into hematopoietic progenitors by cell extract treatment. Here, we report an efficient strategy to generate erythroid progenitors from hESCs using cell extract from human fetal liver tissue (hFLT) with cytokines. Human embryoid bodies (hEBs) obtained of human H1 hESCs were treated with cell extract from hFLT and co-cultured with human fetal liver stromal cells (hFLSCs) feeder to induce hematopoietic cells. After the 11 days of treatment, hEBs were isolated and transplanted into liquid medium with hematopoietic cytokines for erythroid differentiation. Characteristics of the erythroid cells were analyzed by flow cytometry, Wright-Giemsa staining, real-time RT-PCR and related functional assays.

**Results:**

The erythroid cells produced from hEBs could differentiate into enucleated cells and expressed globins in a time-dependent manner. They expressed not only embryonic globins but also the adult-globin with the maturation of the erythroid cells. In addition, our data showed that the hEBs-derived erythroid cells were able to act as oxygen carriers, indicating that hESCs could generate functional mature erythroid cells.

**Conclusion:**

Cell extract exposure with the addition of cytokines resulted in robust erythroid -like differentiation of hEBs and these hEBs-derived erythroid cells possessed functions similar to mature red blood cells.

## Background

Red blood cells (RBCs) have been utilized as the treatment for severe blood loss and hematopoiesis study; but their clinic application has been constrained by limited quantities and compatibility issues. The availability of hESCs offers a great opportunity to produce large quantities of erythroid cells *in vitro *for transfusion, and to provide additional knowledge to the field of erythropoiesis. Previous studies have generated primitive erythroid cells from hESCs by embryoid body formation and stromal cell co-culturing [1-7]. However, the risk of mouse-related diseases and the low differentiation efficiency of hESCs are major limitations of the clinical application of this study.

Recently, we have established a method to produce relatively large number of human hematopoietic cells from hESCs, via a human-derived induction system, by using hFLSCs feeder cells and cell extract of hFLT. Use of this culture method enabled the production of 32.73% CD34^+ ^from treated hEBs after 11 days of culture. More importantly, hEBs-induced hematopoietic cells predominantly yielded erythroid precursors when seeded on methylcellulose [8]. Based on the above results, we isolated the 11- day hEBs from the co-culture system and transplanted them into liquid medium for a 16-day extending culture. During the 16-day culture, cytokines are used to first promote the proliferation and subsequently used for the maturation of erythroid precursors. This culture method enabled the production of about 5 × 10^6 ^fully differentiated erythroid cells from about 5 × 10^4 ^hEBs. The erythroid cells morphologically resembled fetal liver-derived erythroblasts, they mainly expressed embryonic hemoglobin and could be enucleated. Our results show that induction of hESCs into mature erythroid cells *in vitro *is possible by treatment with cytokine-supplemented cell extract.

## Results

### The effects of hFLT cell extract treatment on hEBs

**Figure 1:**
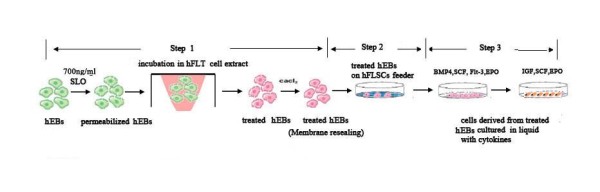
**The inducing system to produce erythroid cells from hEBs**. Step1. hEBs were treated by hFLT cell extract. Step2. The treated hEBs were co-cultured with hFLSCs feeder. Step3. Erythroid differentiation of hEBs in liquid medium with cytokines.

After culture on low-attachment plates for about 24 hours, flat hESCs differentiated into typically round hEBs. The permeabilization of hEBs was analyzed with the streptolysin-O (SLO) assay. In a previous study, we found that most hEBs could be labeled with Texas Red containing 700 ng/ml SLO [8], therefore we chose to incubate hEBs with 700 ng/ml SLO for 50 min in this experiment. After incubation, the permeabilized hEBs were exposed to hFLT cell extract. To reseal cellular plasma membranes, cells were then cultured in IMDM containing 10% fetal calf serum (FCS) and 2 mM CaCl_2_. (Figure 1)

### The capacity of erythroid-like development of hEBs

In a previous study, we found that cell extract treatment could influence differentiation of hEBs but only hFLT cell extract treatment could improve hematopoietic differentiation of hEBs [8]. This experiment provided an opportunity to conduct a large-scale investigation of hESCs-derived erythropoiesis after hFLT cell extract treatment. Firstly, we treated hEBs with hFLT cell extract as described previously [8]. Then the treated hEBs were co-cultured on the hFLSCs feeder in hEBs differentiation medium for the hematopoietic differentiation, and the untreated hEBs were culture in the same condition as a control.

**Figure 2:**
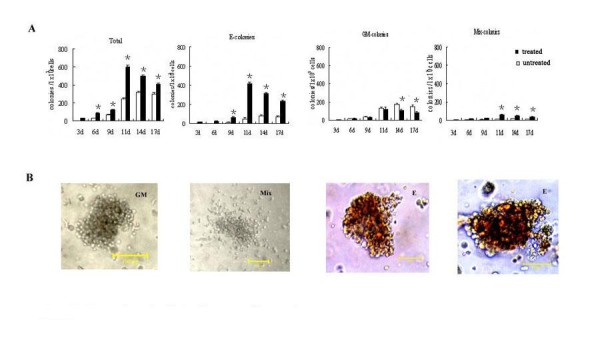
**Generation of colonies from co-culture system**. (A)Statistical analysis for the different kinds of blast colonies generated from treated and untreated hEBs at different day. The data represent the mean ± SEM from three experiments. *, P < 0.05 when colonies generated from treated hEBs were compared with colonies generated from untreated hEBs at the same day. (B)Typical CFU-GM, CFU-Mix, and CFU-E colonies derived from hEBs after being seeded in methyl-cellulose. Scale bar, 100 um.

To examine the capacity for erythroid development of hEBs, the cells were analyzed by hematopoietic colony assays, and colonies were scored according to their cellular morphology. Our results showed that, for untreated hEBs, the colony-forming cells (CFCs) were first found in the day-6 hEBs and various types of hematopoietic CFCs increased rapidly afterwards, including colony-forming units-granulocyte-macrophage (CFU-GM), colony-forming units- macrophage (CFU-M), colony-forming units-erythroid (CFU-E) colonies, and they reached a peak in day-14 hEBs. As shown in Figure 2, most of the hematopoietic colonies were CFU-GM. At the peak (day 14), about 1 × 10^5 ^untreated hEBs-derived cells generated 315.2 ± 25.3 hematopoietic colonies, of which about 55% were CFU-GM and about 22% were erythroid bursts. For hFLT cell extract treated hEBs, hematopoietic colonies were first detected in day-3 hEBs and they reached a peaked on day 11. The number of colony-forming cells produced from the treated hEBs was much greater than that from untreated. At the peak, about 1 × 10^5 ^untreated hEBs-derived cells could generate 655.2 ± 31.3 hematopoietic colonies. Interestingly, hFLT cell extract exposure had a greater effect, both number and frequency, on erythroid colony formation of hEBs. The frequency of CFU-E colonies reached 62% on day 11 of hEBs (Figure 2). Altogether, these data suggested a supportive role of hFLT cell extract on the erythroid lineage development at the clonal level.

### The effects of hFLT cell extract treatment on gene expression

**Figure 3:**
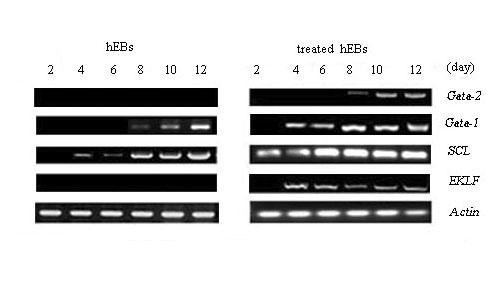
**The gene expression of hEBs**. RT-PCR analysis the erythropoietic markers (*Gata-2, Gata-1, SCL, EKLF*) of treated and untreated hEBs at different days.

To understand the effects of hFLT cell extract treatment on gene expression, we investigated the erythroid associated genes of hEBs at different days of co-cultures including *SCL*, *Gata-2, Gata-1, EKLF *by RT-PCR. We found that the exposure to hFLT cell extract could promote the erythroid associated gene expression (Figure 3). After incubation with hFLT cell extract, *SCL *was first detected as early as day 2 and *Gata-1 *was expressed by day 4, this data confirmed our previous report [8]. The *EKLF *mRNA was detectable by day 4 of differentiation, and its expression was persistently high throughout the remaining of time course. *Gata-2 *was obviously expressed by day 8 and up-regulated from day 10. However, for untreated hEBs, *SCL *could be first detected by day 4 and *Gata-1 *could be found by day 8 at a low level. The other erythroid-associated genes that were tested were difficult to detect before day 12 in the untreated group.

### Production of red blood cells from hEBs

**Figure 4:**
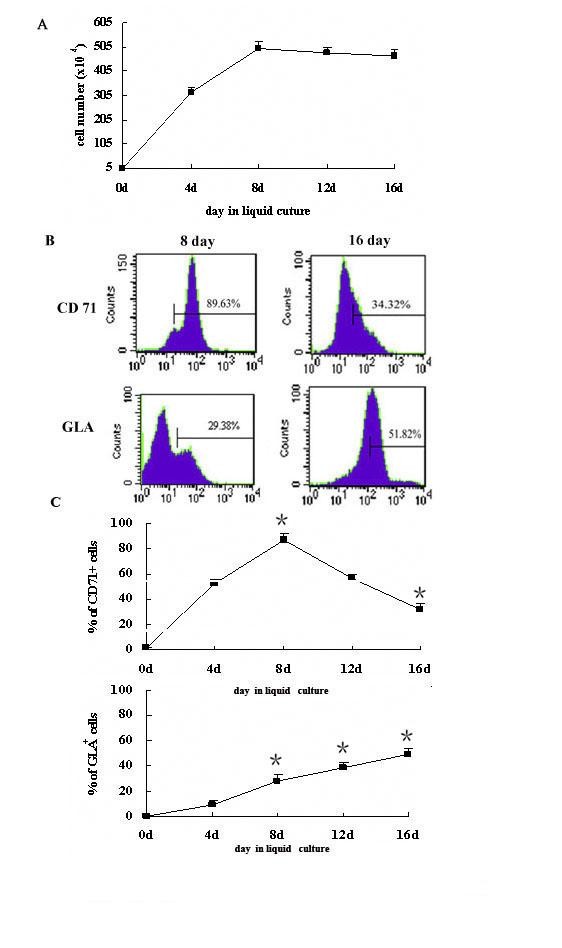
**The production of erythroid cells from treated hEBs**. (A)Amplification of erythroid cells in liquid culture.50000 hEB cells from co-culture could yield about 5000,000 erythroid cells in liquid medium.(B) Analysis of erythroid differentiation of treated hEBs by flow cytometry. Erythroid cells derived from treated hEBs were seeded in liquid medium and tested for expression of CD71 (the marker of early erythroid), GLA (the marker of matuered erythroid) cells by flow cytometry at different inducing times. (C)Statistical analysis for the cell surface antigen expression of hEBs-derived erythroid cells in liquid medium. The data represent the mean ± SEM from three experiments. *, P < 0.05 when compared with the average expression of CD71 or GLA at day 4.

Our data confirmed that hEBs treated with hFLT cell extract possessed a good capacity for erythroid lineage development, and the 11-day hEBs could yield the greatest number of erythroid colonies. To generate a large-scale of erythroid cells from hESCs, we isolated the 11-day treated hEBs and plated cells in liquid medium. Since bone morphogenetic protein-4 (BMP-4) and Flt-3 ligand (Flt-3l) could promote expanding capacity of hematopoietic cells, so cells were incubated with BMP4 (20 ng/mL), stem cell factor (SCF, 100 ng/mL), erythropoietin (EPO, 2 U/mL) and Flt-3l(20 ng/mL) from day 1 to day 8. Insulin like growth factor-1 (IGF-1) was necessary for normal erythroid differentiation[9,10], so erythroid progenitors cells were stimulated by incubation with IGF-1 (20 ng/mL), SCF (100 ng/mL), EPO (4 U/mL)from day 9 to day 16. The number of cells was counted during the 16-day liquid culture period. The results showed that the cells expanded rapidly from day 1 to day 8 and the number of cells increased from 50,000 to 5000,000 per well of 24-well plate. However, the cell numbers did not increase from day 9 to day 16. So about 50,000 hEB cells could yield about 5000,000 erythroid cells during a 16-day incubation, which implied a 100-fold amplification of the erythroid cells produced from the EB cells. (Figure 4A).

To monitor the differentiation of hEB cells into erythroid cells, CD71 (transferrin receptor) and glycophorin A (GLA) antigens were examined by flow cytometry assay throughout the culture. At the beginning of the liquid culture, CD71^+ ^cells were detected at a low lever and GLA^+ ^cells could not be detected at all (data not shown). CD71^+ ^cells increased rapidly from day 1 to day 8, and reached 89.63% of their peak level at day 8 and subsequently decreased. About 34% of the cells were CD71^+^cells at the day 16. In contrast, however, GLA^+ ^cells expanded significantly from day 8 to day 16. GLA^+ ^cells could not reach 30% at the day 8, but peaked 51.82% at the end of culture (Figure 4B, C)

### Analysis of hEBs-derived erythroid cells

**Figure 5:**
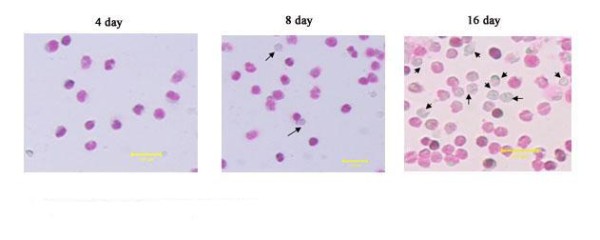
**RBCs obtained in liquid medium**. The erythroid cells derived from hEBs were stained by Wright-Giemsa reagents at different day (the arrows directed enucleated cells). Scale bar, 100 um.

To further characterize the erythroid cells derived from hEBs, we stained cells with Wright-Giemsa reagents and compared them with RBCs derived from human fetal liver. We found that hFLT cell extract treatment plus cytokines promoted enucleation of hEBs-derived erythroid cells. As shown in figure 5, the enucleated hEBs-derived RBCs were first detected at day 8 in liquid medium and approximately 20% to 35% of erythroid cells were enucleated at day 16.

**Figure 6:**
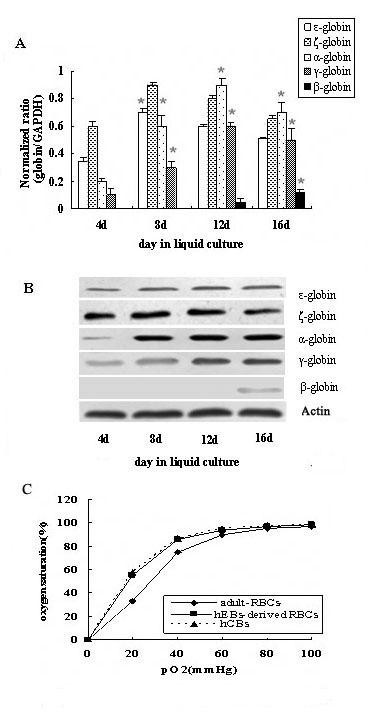
**The feature of hEBs-derived erythroid cells**. (A) Real-time PCR analysis of the globins gene expression of erythroid cells generated from hEBs after 4, 8, 12 or 16 days of liquid culture. *, P < 0.05 when compared with the average expression of ζ, ε, α, γ globins at day 4 and β globin at day 12. (B) Western blotting analysis of the globins protein expression of erythroid cells generated from hEBs at different day. (C) Functional assays of hEBs-derived erythroid cells. Oxygen dissociation curves of RBCs derived from hEBs, hCB or adult blood.

We next examined globin expression patterns of these erythroid cells generated from hEBs after 4, 8, 12 or 16 days of culture by real-time PCR(Figure 6A). Then we found that the cells derived from treated hEBs expressed embryonic globins (ζ, ε, α and γ) but not adult globins (β) at the beginning of culture. The expression of ζ and ε-globins mRNA increased rapidly from day 1 to day 8, subsequently peaked at day 8 and then decreased. The expression of α and γ-globins mRNA peaked at day 12. In contrast, the expression of β-globin was first detected at day 16 at a low level.

To further observe the expression pattern of globins, we analyzed hEBs-derived erythroid cells at different days by western blotting. We found ζ, ε, α and γ globins proteins were detected by day 4 and β-globin protein could be found at day 16 (Figure 6B), which confirmed that erythroid cells generated from hEBs possessed the capacity to express the adult globin at the end of culture. These data indicated that globins expression of erythroid cells generated from hEBs might begin to switch from the embryonic to the adult type at day 16.

### Functional analysis of hEBs-derived erythroid cells

To assess the function of the hEBs-derived erythroid cells, we measured oxygen dissociation of hEBs-derived erythroid cells and compared them with human cord blood (hCB) and human adult peripheral blood. As shown in Figure 6C, hEBs -derived erythroid cells displayed an oxygen dissociation curve similar to that of hCB, but shifted to the left as compared to the curve of human adult peripheral blood.

## Discussion

In this study, we first established a system to induce the production of a large number of erythroid cells from hESCs utilizing four standard procedures. In the first and second steps, the hEBs were treated with the cell extract from 14-weeks fetal liver and co-cultured with hFLSCs feeders. Cell extract treatment is a novel trans-differentiation strategy that can convert a somatic cell type into another type [11-15]. We recently reported a new method to promote the differentiation of human stem cells toward hematopoietic lineages by the treatment with cell extract of hFLT [8]. The fetal liver is a very important organ of human hematopoiesis, and can generate not only transplantable hematopoietic stem cells, but also enucleated RBCs. We therefore speculated that hFLT cell extract treatment may be advantageous for erythroid line development as well. In the current study, we isolated hEBs from co-cultures after treatment with hFLT cell extract and examined their capacity for the erythroid development at different period of time. At same time the untreated hEBs were isolated from co-cultures as a control. We found that the treated hEBs in the co-culture system could express the erythroid associated genes and give rise to erythroid lineage colonies in semisolid medium, whereas the untreated hEBs did not express erythroid associated genes and mainly yielded CFU-GM with the same experimental conditions. Thus, our data provided strong evidence which shows that treated hEBs-derived hematopoiesis mainly generated erythroid cells.

Furthermore, we isolated the treated hEBs from co-cultures and induced them into erythroid cells in two -phases. At the beginning of the culture, addition of BMP4, Flt-3l, SCF, and EPO resulted in a rapid increase in cell numbers and an accumulation of differentiated cells. CD71 was used as a marker for early erythroid cells and GLA used as a marker for mature erythroid cells. During the first stage of liquid culture, we observed an obvious increase of CD71^+ ^cells and a slight increase of GLA^+ ^cells, which implied the generation and expansion of early erythroid cells from hEBs. From day 9 to day 16, stimulated by IGF-1, SCF and a high dose of EPO, most of the cells had characteristics of mature erythroid cells. During this stage we observed a peak in the percentage of GLA^+ ^cells, but the percentage of CD71^+ ^cells decreased at the end of culture.

To further understand the erythropoiesis of hESCs, we examined globins expression of erythroid cells from hEBs. According to our previous study, erythroid cells derived from hEBs only expressed embryonic globins but not adult globin [5]. However, in this study we found that hEBs-derived erythroid cells expressed both the embryonic and adult globins at the end of culture. One possible explanation for this result relates to the complex process of mammalian erythropoiesis. In the primitive hematopoiesis wave, blood islands in the yolk sac transiently generate nucleated RBCs. In the later stage of development, the fetal liver is the primary site for production of transplantable hematopoietic stem cells and enucleated RBCs. Definitive hematopoiesis ultimately shifts to the bone marrow, the site for production of life-long adult-type hematopoiesis[2]. Erythrocytes from different hematopoietic sites expressed different types of globins. Yolk sac-derived erythrocytes express only embryonic globins, and erythroblasts produced in fetal liver express embryonic globins and a small amount of adult globin. Erythroblasts from bone marrow primarily expressed adult globin. In our inducing system, hEBs were treated with hFLT cell extract and co-cultured with hFLSCs. It is possible that this condition mimics the environment of the fetal liver and results in similar globin expression of the hEBs-derived erythroid cells as with fetal liver-derived erythroid cells.

A critical issue for clinical application of hESCs is whether they can generate functionally mature progenies. In our system, enucleated RBCs could first be observed at day 8 of liquid culture. The number of enucleated RBCs increased rapidly from day 12 to 16. The erythroid cells produced from treated hEBs morphologically resembled RBCs from human fetal liver. Capability of carrying oxygen is an important function of RBCs. To determine if hEBs-derived erythroid cells possessed the same function as normal RBCs, we analyzed the function of hEBs-derived erythroid cells and found that the oxygen dissociation curve of hEBs-derived erythroid cells shifted to left compared to the curve of human adult blood. To our knowledge, the oxygen dissociation curve of hCB was shifted left when compared to that of human adult peripheral blood [16]. Our results implied that hEBs-derived erythoid cells were also able to function as oxygen carriers and the oxygen dissociation pattern of these cells was more similar to fetal blood cells than adult blood cells. Thus RBCs derived from hEBs in our system may act as an alternative resource of RBCs from blood.

## Conclusion

Hematopoietic differentiation of hESCs has been achieved by using a variety of experimental approaches[1,2]. In addition, the successful derivation of RBCs from hCB has been achieved by many published assays[3,4]. However, there are only a few published reports on erythroid differentiation from hESCs. Trans-differentiation of a somatic cell type into another cell type would be beneficial for producing replacement cells for potential therapeutic applications. In this study, we first reported that fetal liver cell extract-based treatment induced the differentiation of hESCs into RBCs safely and efficiently. Our method provides a useful tool for studying the molecular mechanisms of hematopoietic development, and will be valuable in the production of RBCs for transfusion.

## Methods

### Cell culture

hESC H1 cells were obtained from WiCell Research Institute, Inc. (Madison, WI, USA). To prevent cells from differentiating, cells were co-cultured with irradiated (20 cGy) mouse embryonic fibroblast (MEF) cells and maintained in knock-out Dulbecco's modified Eagle's medium (DMEM) supplemented with 20% serum replacer (SR), 1% nonessential amino acids (NEAA), 1 mM L-glutamine (all from Invitrogen Corporation, Carlsbad, CA, USA), 0.1 mM β-mercaptoethanol (Sigma-Aldrich, USA) and 4 ng/ml human basic fibroblast growth factor (bFGF) (R&D Systems, USA).

The hFLSCs were isolated and cultured as previously described [17].

### Preparation of cell extract

The hFLT cell extract was prepared, as described previously [18,19], from 15-week human aborted fetal liver tissue obtained with informed consent. Briefly, the hFLT cell extract was prepared as follows: cells were washed in phosphate-buffered saline (PBS) and resuspended in cell lysis buffer. Cells were lysed with a dropping pipette and centrifuged at 15,000 × *g *for 15 minutes at 4°C. The supernatant was removed, filtered and stored at -80°C.

### Inducing erythroid differentiation of hEBs

#### Step 1. Cell Extract treatment of hEBs

Undifferentiated hESCs were harvested at 80% confluence after collagenase IV digestion and transferred to 10-cm low cell-binding dishes to form hEBs in hEBs medium containing KO-DMEM supplemented with 20% FBS, 1% NEAA, 1 mM L-glutamine, and 0.1 mM β-mercaptoethanol. The hEBs were then treated with hFLT extract. Briefly, the 2-day hEBs were treated with SLO in Ca^2+-^Mg^2+^-free Hanks' balanced salt solution (Gibco-BRL, Invitrogen) for 50 minutes at 37°C. About 100 μl of hFLT cell extract containing an XX ul or % ATP-generating system (1 mM ATP, 1 mM GTP, 1 mM NTP, 10 mM phosphocreatine, and 25 μg/ml creatine kinase, Sigma) were added to replace the SLO and incubated for 60 minutes at 37°C. To reseal plasma membranes CaCl_2 _(2 mM) was added to the hESCs culture medium, and cells were cultured overnight at 37°C.

#### Step 2. hFLSCs feeder co-culture

The treated hEBs were cultured with hFLSCs feeder in hEBs differentiation medium for 11 days containing 80% IMDM (Invitrogen), 30% FBS (Invitrogen), 1% NEAA, 2 mM L-glutamine, 0.1 mM beta-mercaptoethanol. After some days of culture, cells were collected and analyzed to identify erythroid differentiation. Total RNA was isolated from hEBs at different days of co-culture using the following the manufacturer's protocol. We analyzed mRNA expression for markers of erythroid cells by RT-PCR. In addition, the EB cells at different days of co-culture were collected and seeded in MethoCult GF -H4434 semisolid medium (Stem Cell Technologies, USA) to determine their capacity for erythroid development.

#### Step 3. Erythroid differentiation of hEBs in liquid medium with cytokines

The hEBs that possessed the greatest capacity for erythroid development were dissociated into single-cell suspensions by collagenase IV treatment. Cells were induced into erythroid cells by two phases: from day 1 to day 8, cells were cultured in medium composed of IMDM supplemented with 30% FBS, BMP4 (20 ng/mL), SCF (100 ng/mL), EPO (2 U/mL), and Flt-3l(20 ng/mL); from day 9 to day 16, cells were cultured in medium composed of IMDM supplemented with 30%FBS, IGF-1 (20 ng/mL), SCF (100 ng/mL), EPO (4 U/mL). About 1 to 2 mL fresh medium was added to each plate every 2 to 3 days,.

### RT-PCR

The gene expression of hEBs was examined by RT-PCR. All PCR reactions were performed as follows: 95°C for 5 min; 94°C for 40 s; annealing at various temperatures for 40 s, 72°C for 40 s (25 cycles); 72°C for 10 min, 4°C for 5 min. The forward and reverse primers are shown in Table 1:

**Table 1:** Primer sequences for RT-PCR

N**ame**	Primer sequence (sense)	Primer sequence (antisense)
*Gata-1*	CAGGTACTGCCCATCTCTAC	TCTGGCTACAA G AGGAGAAG
*Gata-2*	AAGGCTCGTTCCTGTTCAGA	GGCATTGCACAGGTAGTGG
*SCL*	GCTGGCTTTTCTGTTTCCTG	TGACA ACCCCAGGTCTTA GG
*EKLF*	CGGACACACAGGATGATCTC	GGCTGGTCCTCAGACTTCAC
*β-Actin*	GATCCACATCTGCTGGAAGG	AAGTGTGACGTTGACATCCG

### Clonogenic progenitor cell assay

Hematopoietic clonogenic assays were performed in 35-mm low adherent plastic dishes using MethoCult GF -H4434 semisolid medium (Stem Cell Technologies, USA) consisting of 1% methylcellulose, 30% FBS, 1% bovine serum albumin (BSA), 50 ng/mL stem cell factor, 20 ng/mL granulocyte-macrophage colony-stimulating factor (GM-CSF), 20 ng/mL IL-3, 20 ng/mL IL-6, 20 ng/mL granulocytecolony-stimulating factor (G-SF), and 3 units/mL erythropoietin(EPO). After culturing for 12-14 days, colonies were scored according to their cellular morphology.

### Real-Time PCR

The expression of globins in erythriod cells was examined by real-time RT-PCR using the SYBR Green PCR Master Mix. All PCR reactions were performed as follows: 95°C for 5 min; 94°C for 40 s; annealing at various temperatures for 40 s, 72°C for 40 s (25 cycles); 72°C for 10 min, 4°C for 5 min. The forward and reverse primers used were described in Table 2 as follows:

**Table 2:** Primer sequences for Real-time PCR

Name	Primer sequence (sense)	Primer sequence (anti-sense)
*ζ-globin*	GCTCAGGCCGAGCCCATTGG	TAGCGGTACTTCTCAGTCAG
*ε-globin*	GGAGAGTCCATTAAGAATCTA	CTGTGAATTCATTGCCGA AGT
*α-globin*	CGGTCAACTTCAAGCTCCTAAG	CCGCCCACTCAGACTTTATT
*β-globin*	TACATTTGCTTCTGACACAAC	ACAGATCCCCAAAGGAC
*γ-globin*	CTTCAAGCTCCTGGGAAATGT	GCAGAATAAAGCCTACCTTGAAAG
*GAPDH*	CTGACTTCAACAGCGACACC	TGCTGTAGCCAAATTCGTTGT

### Western blotting

The cell extract was subjected to sodium dodecyl sulfate-polyacrylamide gel electrophoresis (SDS-PAGE) and transferred onto a polyvinylidene difluoride membrane (Millipore, Bedford, MA). After blocking in TBST containing 5% dry-fat skim milk, the membrane was incubated with anti-globin antibodies (Santa Cruz Biotechnology), followed by HRP-labeled goat anti rabbit IgG. The protein bands were visualized by enhanced chemiluminescence (ECL; Pierce).

### Flow cytometric analysis

The trypsinized individual cells were incubated with the following FITC-conjugated and PE-conjugated monoclonal antibodies: anti-human CD71, anti-human glycophorin A (B&D Biosciences, USA) for 30 minutes at 4°C. Cells were washed with PBS for three times, and analyzed by flow cytometeric analysis using the FACSCalibur (Becton-Dickinson, Mountain View, CA, USA).

### Wright-Giemsa staining

Cells were dropped onto slides and fixed for 20 minutes in 4% paraformaldehyde and were stained with Wright-Giemsa reagents (Fisher Scientific) following manufacturer's instructions.

### Functional Assays for Erythroid Cells

The oxygen binding ability of hESCs-derived erythroid cells and human adult blood was measured with a Hemox analyzer, as reported previously [20,21].

### Statistical analysis

Results were expressed as means ± SEM. Statistical significance was determined using Student t- test. Results were considered significant at P < .05.

## Authors' contributions

YXL participated in the design of the study and carried out most of the experiments. XTP and WY participated in the coordination and design of the study. WY also helped in drafting the manuscript. LJ helped in cell culture and helped draft the manuscript. XN helped with RT-PCR. All authors read and approved the final manuscript.
